# Application of Exergy-Based Fault Detection in a Gas-To-Liquids Process Plant

**DOI:** 10.3390/e21060565

**Published:** 2019-06-05

**Authors:** Sarita Greyling, Henri Marais, George van Schoor, Kenneth Richard Uren

**Affiliations:** 1 School of Electrical, Electronic, and Computer Engineering, Faculty of Engineering, North-West University, Potchefstroom 2531, South Africa; 2 Unit for Energy and Technology Systems, Faculty of Engineering, North-West University, Potchefstroom 2531, South Africa

**Keywords:** fault, detection, isolation, exergy, petrochemical

## Abstract

Fault detection and isolation (FDI) within the petrochemical industries (PCIs) is largely dominated by statistical techniques. Although a signal-based technique centered on exergy flows within a process plant was proposed, it has only been applied to single process units. The exergy-based scheme has not yet been applied to process plants that feature at least a single recycle stream. The Tennessee Eastman process (TEP) is commonly used as an FDI benchmark process, but due to obfuscation, the TEP cannot be directly implemented in a commercial process simulator. Thus, application of FDI techniques to proprietary processes will require significant investment into the implementation of the FDI scheme. This is a key impediment to the wide-spread comparison of various FDI techniques to non-benchmark processes. In this paper, a gas-to-liquids (GTL) process model is developed in Aspen HYSYS^®^, and the model’s performance is validated. The exergy-based FDI technique is applied to the GTL process while the process is subjected to carefully selected faults. The selected faults aim to affect several process units, and specifically, the resultant recycle stream of the GTL process is considered. The results indicate that even though the exergy-based technique makes use of fixed thresholds, complete detection and isolation can be achieved for a list of common process faults. This is significant since it shows, for the first time, that the exergy-based FDI scheme can successfully be deployed in processes with recycle streams.

## 1. Introduction

The world’s total energy consumption continues to rise with the total primary energy demand (TPED) expected to reach 17,500 million tonnes of oil equivalent by 2050 according to the World Energy Index of the IEA. Based on predictions by the IEA [1], petrochemical industries (PCIs), specifically plastics manufacturing, will see the largest increase in demand by 2050. However, currently transportation fuels account for the bulk fraction of oil demand globally with Asian countries, which are seen as the largest consumers [2]. For this reason, the safe and efficient operation of petrochemical process plants (PCPPs) is critical. These plants represent some of the most complex structures encountered in engineering. Additionally, these plants also represent a significant safety risk and environmental hazard. In order to mitigate these risks, the fields of abnormal situation management (ASM), condition monitoring (CM) and fault detection and isolation (FDI) are deployed.

Commonly, FDI is classified as either being model-based or data-driven [3,4,5], with the primary distinction being the availability of an analytic model. Although not impossible, the derivation of analytic models for PCI plants is considered to be challenging. An example of the complexity associated with the derivation of analytic models is provided in [6] for the Tennessee Eastman Process (TEP). However, in a review series, Gao et al. [7,8] introduced the so-called signal-based diagnosis which makes use of measurement signals rather than analytic models of the plant. Signal-based diagnosis is still considered to be model-based since the *normal* signal is known a priori.Data-driven methods, on the other hand, require large amounts of data processing but without any a priori knowledge. Excluding qualitative knowledge-based FDI systems (expert systems) [8], the remaining data-driven approaches can be classified as being either primarily statistical or based on machine learning approaches. Within the PCIs, data-driven methods are particularly dominant. Qin et al. [9] applied several statistical process monitoring (SPM) schemes to an industrial polymer film process. Other studies applied principal component analysis (PCA) techniques to continuous stirred tank reactors (CSTRs) [8,10] and plug-flow reactors [11], and the use of mathematical models to generate data is also not uncommon [8,12]. A key advantage of model-based FDI is that the model can be used to mathematically prove the extent of the technique. As such, a model-based FDI would be ideal for high-risk applications such as petrochemical processes.

Given the dominance of data-driven FDI within the PCI and the advantages afforded by model-based FDI techniques, the development of a model-based FDI technique for the PCIs is warranted. Several authors have suggested a signal-based FDI that measures the energy flows within a process plant. Du Rand [13] applied an entropy-enthalpy approach to the Brayton cycle of a nuclear power plant, and Marais et al. [14] showed that condition monitoring of a CSTR from an energy perspective could be accomplished. In later work, Marais [15] showed that exergy could successfully be used to perform FDI for an auto thermal reformer (ATR).

In this work, the development of the gas-to-liquids (GTL) process provides a suitable way to enable the exploration of the the exergy-based FDI scheme’s effectiveness in terms of more complex processes. Since it is clear that no single FDI scheme will perform perfectly in all conditions [16], the development of hybrid approaches is required. Essentially, hybrid FDIs combine existing techniques to optimally exploit the various advantages offered by the constituent techniques. A typical example of a hybrid FDI is the combination of statistical and machine learning techniques [17]. Combinations of signal- and observer-based techniques have also proven to be successful when applied to a classical two-tank system [18]. As noted in [16], a simple combination of techniques will not necessarily provide superior results. Based on conjecture, a combination of model-based and data-driven FDI schemes should provide significant improvements in terms of both FDI performance and reliability. In [19] the application of the exergy-based FDI scheme (considered to be a hybrid scheme) to a process containing recycle streams was identified as an area of future work. Indeed, the ATR plant used by [19] did not include any recycle streams. Thus, the applicability of the exergy-based technique to PC plants that include recycle streams remains lacking. Additionally, given the limited examples of model-based FDI in the PCIs, the limitations of existing implementations and the applicability of new methods need to be explored.

The novelty of this work is the determination of the applicability of an exergy-based FDI scheme to a GTL process. To this end, the authors propose the development of a GTL process in Aspen HYSYS^®^ (a dynamic chemical process simulator). The developed model (available online [20]) is only operated in the steady state to allow initial exploration of the exergy-based FDI scheme’s performance. However, the model lends itself to dynamic simulation. The implementation of the exergy-based FDI scheme within the process simulator allows the technique to be easily applied to existing proprietary processes with minimal effort. The availability of a process model or benchmark in a commercial process simulator specifically with the aim of supporting FDI scheme development allows the comparison of various FDI approaches in a transparent manner. Additionally, and more importantly, since many proprietary processes have already been modeled in process simulations, the benchmarking of existing FDI approaches to these processes is viable.

The development and validation of the model is presented in Section 2.2. The proposed exergy-based FDI scheme is detailed in Section 2.3, and the results are presented in Section 3. A discussion of the results and concluding remarks are provided in Section 4.

## 2. Methodology

In this section, justification for the development of a suitably complex process model is provided by means of a brief literature review. The identified process model lends itself to the implementation of a hybrid FDI scheme that is fundamentally based on the concept of exergy characterization. The development of the GTL plant model is presented, followed by the details of the proposed exergy-based FDI scheme. The GTL model is developed from the literature, and its performance is validated. It should be noted that the focus of the model is on the technical accuracy of the process itself and not on a techno-economic optimal operating point per se.

### 2.1. Process Selection Rationale

Unfortunately, the varied use of differing case studies and academic models complicates the direct comparison of various FDI techniques within the PCIs, especially when the nature of the processes is fundamentally different. One way of addressing the issue is to use a known benchmark process. The TEP was initially developed as benchmark control problem [21] but later became the defacto FDI benchmark process [22]. Jiang et al. [23] showed that typical PCA algorithms might fail to select the contributing components when applied to the TE process and proposed the sparse PCA selection (SPCS). In [24], the TE process was again used to illustrate the advantages of a moving window PCA and fuzzy logic hybrid approach. In a review by [25], several other applications of FDI to the TEP were also presented. Recently, machine learning (ML/AI) approaches have also been applied to the TEP [26], including deep learning approaches [27]. Hybrid techniques, such as those of [17], have also been successfully applied to the TEP. This seems to suggest that the TEP is ideally suited as a FDI benchmark.

However, considering the TEP specifically, many authors have made use of the data sets published by Russel et al. [28] with the original FORTRAN formulation adapted for use in Matlab Simulink [29]. Bathelt et al. identified several deviations in later Simulink results and updated the underlying process code [30]. Only recently has a Modellica model been developed by [31] that can be controlled from Matlab Simulink. Although the work by [31] is more usable, the presence of a TEP in commercial process simulators remains lacking. In [32], the applicability of Aspen One as a commercial process simulator was claimed. However, the formulations of the obscured components (A through F) in the original TEP formulation were never justified, and since no details of the Aspen One flowsheets were provided, duplication of the results in Aspen HYSYS^®^ is impossible. Due to the additional complexities introduced by the obfuscation of process parameters in the original TEP description, implementation in commercial process simulators (such as Aspen HYSYS^®^) is a complex task. However, the advantages of an accessible benchmark process with representative complexity could provide significant benefits to the FDI community.

The gas-to-liquids process is used either partly (generating components) or in totality (natural gas to transportation fuels) in many industrial processes [33]. Most of the models in scientific literature focus on the techno-economic aspects of the GTL process [34] and, as such, are not ideally suited to FDI endeavors. In order to address the complexity of comparing various FDI approaches within a PC context, the development of a benchmark process model is proposed. The GTL process, of which the ATR is the first process unit [19], is seen as being sufficiently representative from a complexity perspective but has also been sufficiently documented to allow for implementation in a commercial process simulator such as Aspen HYSYS^®^. In this paper, the process model developed aims only to be sufficiently complex (thus containing at least one recycle stream) to evaluate the performance of a exergy-based FDI scheme.

### 2.2. GTL Model Development and Validation

**Figure 1:**
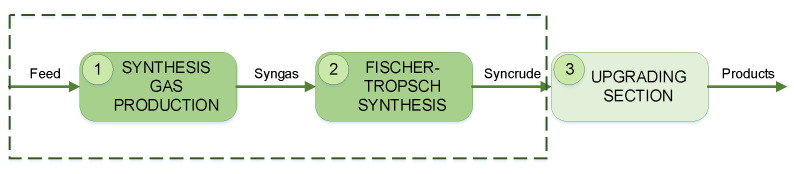
A process diagram of a gas-to-liquids (GTL) process.

A GTL process takes gaseous feedstock (such as natural gas) and transforms it into liquids. A GTL process generally comprises three major sections, as shown in Figure 1.

In the first section, synthesis gas production, natural gas is reformed in order to obtain synthesis gas (syngas). Syngas consists of hydrogen (H_2_) and carbon monoxide (CO), usually in a particular ratio depending on the desired products. Next the syngas is introduced to a Fischer–Tropsch reactor which converts the syngas to a range of hydrocarbons (also referred to as syncrude). The last section upgrades the obtained syncrude to hydrocarbon products of specific chain lengths. Comprehensive details can be found in the works of [35,36,37].

Considering the complexity of the upgrading section, this study only focuses on the syngas production and Fischer–Tropsch synthesis sections that are shown in boxes in Figure 1. It should also be noted that the developed GTL model will be utilized as a representative system for FDI applications only. Therefore the model is based on open literature, and no attempts were made to improve the process in any way.

**Figure 2:**
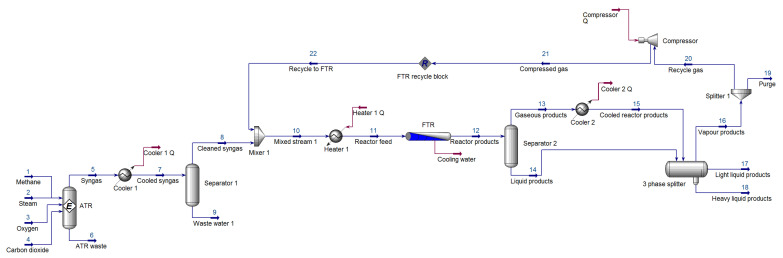
The Aspen HYSYS^®^ process flow diagram of the developed GTL process.

Given the prevalence of the GTL process, a variety of technologies can be utilized for syngas production section. When reviewing existing literature, such as [37], most researchers implement auto thermal reformers (ATRs). ATRs have several advantages [38] amongst which are their economy of scale, smaller footprint, and faster start-up and load transitions. Other authors have also suggested that ATR shows the most promise in terms of GTL processing [39]. Given the suitability of the ATR for use in a GTL process that is fed by natural gas (mainly methane), and the advantages it offers at a large-scale for single process streams, this study implements an ATR. De Klerk [40] emphasized the importance of the temperature and composition (ratio of H_2_/CO) of the syngas produced. For the specific GTL configuration considered (shown in Figure 2), it is expected that the temperature should vary within the range of 1020–1065 °C with H_2_/CO ≈2.0. In order to produce syngas of adequate temperature and composition, the ATR is fed specific ratios of natural gas, steam, and oxygen. It has been shown that oxygen greatly affects the syngas temperature, and in some studies, such as [41], a carbon dioxide (CO_2_) stream was included to aid in the control of the syngas composition. The produced syngas is then cleaned and fed (at temperatures between 200–240 °C) into the Fischer–Tropsch reactor (FTR). The considered hydrocarbons included C_2_ to C_20_. C_30_ was used to represent hydrocarbons C_21–30_ which exhibit similar properties. The generation of these hydrocarbons followed an Anderson–Schulz–Flory (ASF) distribution, relating closely to the distributions seen in [37,42]. Usually, unreacted components are recycled to be put through the process again, whilst the liquid products are transferred to the upgrading section.

In order to reduce the complexity of the GTL process, the following assumptions and adjustments were made:

1. No pre-reformer was included, since for this study, there was no recycling to the ATR.
2. The ATR’s natural gas feed stream was modeled as pure methane (CH_4_).
3. A carbon dioxide (CO_2_) stream was added to manipulate the syngas composition.

The subsequent sections detail the development and validation of the GTL model.

#### 2.2.1. Autothermal Reformer

In order to model the ATR, an equilibrium reactor in Aspen HYSYS^®^ was used. The three most important reaction equations, as indicated by [36], are the oxidation of methane (1), the steam reforming of methane (2), and the water gas shift reaction (3):

(1) $${\mathrm{CH}}_{4}+1.5O_{2}\;⇌\;CO+2H_{2}O$$

(2) $$CH_{4}+H_{2}O\;⇌\;CO+3H_{2}$$

(3) $$CO+H_{2}O\;⇌\;CO_{2}+H_{2}.$$

As previously mentioned, the feed streams were fed into the ATR in certain molar flow rate ratios. By fixing the methane stream at 8195 kgmole/h, a molar flow rate frequently seen in literature, such as in [37], the steam, oxygen, and carbon dioxide molar flow rates were calculated by using the ratios summarized in Table 1. The simulated syngas was found to be acceptable with a temperature of 1029 °C and a composition of 2.105. A cooler was incorporated to cool the produced syngas to 38 °C. The waste heat generated by the cooler is not used within the process and is essentially returned to the environment. From a plant design perspective, this is inefficient. However, in doing so, the process is kept as simple as possible, and this also does not affect the application of the FDI scheme to the GTL process in any way. Similar arguments hold for the energy streams of compressors and coolers used elsewhere in the process. Using a separator, the excess produced water can be removed, feeding cleaned syngas to the Fischer–Tropsch reactor.

#### 2.2.2. Fischer–Tropsch Reactor

The cleaned syngas needs to be fed to the FTR at a temperature in the range of 200–240 °C. Therefore a heater was included to heat the cleaned syngas (stream 8) from ambient temperature to 210 °C. A plug flow reactor (PFR) was used as the FTR, as [37] suggests that it is representative of a multi-tubular fixed bed (MTFB) reactor. A reactor with a volume of 1000 m^3^ was implemented. Equations (4) and (5) were modeled as kinetic reactions within Aspen HYSYS^®^. Equation (4) describes the Fischer–Tropsch reaction with the stoichiometric coefficients detailed in [37,43]. These researchers made use of a constant chain growth factor α of 0.9. The inevitable production of methane is included and is described using (5):

(4) $$\begin{array}{c} CO+2.1H_{2}\;\to \;\sum_{n=1}^{20}v_{n,1}C_{n}H_{2n+2}+v_{30,1}C_{30}H_{62}+H_{2}O \end{array}$$

(5) $$CO+3H_{2}\;⇌\;CH_{4}+H_{2}O.$$

Next the rate expressions of the equations need to be specified. With so many different kinetic mechanisms presented in literature, the most popular approach seems to be that developed by [44], given by (6) and (7)

(6) $$r_{CH_{4}}=\frac{k_{1}P_{H_{2}}{P_{CO}}^{0.05}}{1+K_{1}P_{CO}}$$

(7) $$r_{CO}=\frac{k_{2}{P_{H_{2}}}^{0.06}{P_{CO}}^{0.65}}{1+K_{1}P_{CO}}.$$

It is important to note that [35,36,37] converted the rate expressions of [44] to more universal units. Table 2 summarizes the values and the units that were used within Aspen HYSYS^®^.

**Figure 3:**
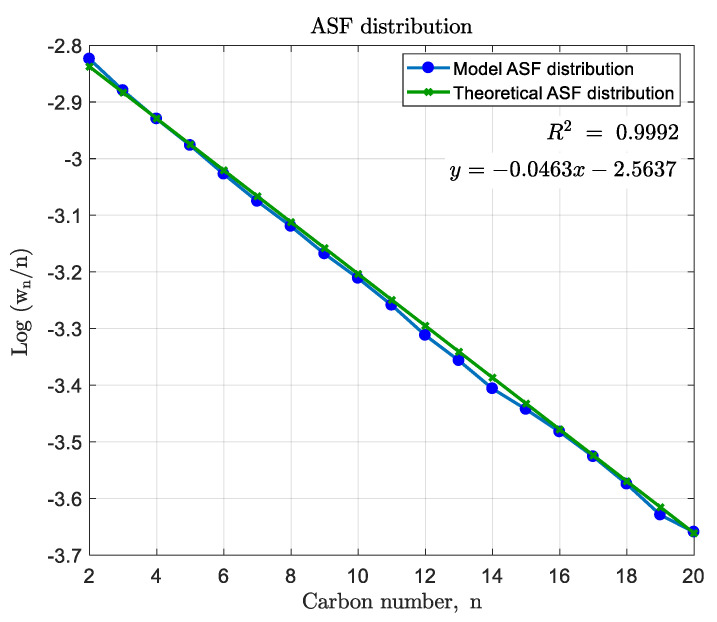
The Anderson–Schulz–Flory (ASF) distribution of the Fischer–Tropsch reactor (FTR) products C_2_–C_20_.

In order to validate whether the model produces the anticipated products, the product distribution was evaluated. This was done by observing the weight fractions of the components in stream 12, not including the recycling stream as of yet. The weight fractions ($w_{n}$) were firstly divided by their corresponding carbon numbers (*n*) and then the logarithm of each was calculated. These log-values were then plotted against their carbon numbers. For the ASF distribution, a straight line with slope log(α) was expected. Consequently for a chain growth probability of α=0.9, the slope was expected to be −0.04576. When plotting the obtained products, as depicted in Figure 3, it was found that the slope was −0.4630. As stated by [37], C_30_ is representative of the lumped components C_21-30_ and should not be included in the distribution plot. The obtained slope deviated by 1.2% from the theoretical slope; therefore, the simulated products were found to be adequate.

With the FTR section simulated and validated, it was possible to develop the remainder of the GTL process. In order to yield the two streams that a multi-tubular fixed bed reactor would produce, a separator was added after the PFR, hence providing a vapor product stream and a liquid product stream. In order to remove some of the unwanted water, the vapor stream (stream 13) was cooled to 38 °C. This cooled stream and the liquid product stream were then fed into a three-phase separator. In such a system, the light and heavy liquid products are usually forwarded to the upgrading section. The vapor products (stream 16) are split into a recycle stream and purge stream (0.8:0.2). The recycled stream is compressed and fed back to the FTR, based on the work of [37]. The connection of the recycle block concluded the simulation effort. Table 3 summarizes the stream information extracted from Aspen HYSYS^®^.

### 2.3. Implementation of Exergy-Based Fault Detection

Exergy is defined as being a quantitative measure of an energy quantity’s usefulness to perform work [45]. Eventually, energy reaches thermodynamic equilibrium with its environment and it can no longer deliver valuable work. The main advantage of using exergy is that it provides a means of quantifying the quality of an energy stream and also the efficiency of any process elements. All processing elements (including transportation elements such as pipes) have an associated efficiency. Should this efficiency change, it would be indicative of some process anomaly. Especially in chemical processes, the total energy contained in a process stream can remain constant while the usefulness declines. Although the total system energy would remain constant, the energy that has been transformed into less useful forms (waste heat for instance) is much easier to account for in exergy terms.

In essence, a system’s exergetic behaviour provides a much more intuitive representation, since exergy can be destroyed. The total exergy of a system, which has no magnetic, nuclear, electric, or surface tension characteristics, is usually expressed as (in specific exergy notation)

(8) $$b_{tot}=b_{kin}+b_{pot}+b_{ph}+b_{ch},$$

where $b_{kin}$ refers to the kinetic exergy, $b_{pot}$ to the potential exergy, $b_{ph}$ to the physical exergy, and $b_{ch}$ to the chemical exergy of the system. Seeing as the physical GTL plant is static, the kinetic and potential exergy can be disregarded, simplifying (8) to

(9) $$b_{tot}=b_{ph}+b_{ch}.$$

Exergy is always evaluated relative to a reference environment (RE). This means that the RE’s intensive properties will determine the exergy. For physical exergy, these include temperature and pressure only and are usually $T_{0}=25$ °C and $P_{0}=101.325$ kPa. The chemical exergy, however, is based on an environment which comprises certain reference elements and intensive properties. Different approaches for the selection and calculation of the standard chemical exergy of these reference elements exist, with the most prominent RE being the one proposed by [46].

Fundamentally, however, the use of exergy as the monitored parameter leverages the structural information contained in the process itself [47]. It has also been shown that exergetic efficiencies could also be used to diagnose the component level under performance in a biomass gassifier [48], and similar work was done by [49,50] pertaining to turbines. This seems to suggest that exergy is well suited to detect component level inefficiencies (degradation or faults) when the system level performance degrades. Indeed, Ref. [51] showed that exergy can be used to determine the efficiency of fired heaters, of which the ATR is a typical example. For many PCI processes, the cyclic nature of the process presents a particularly challenging scenario as feedstocks and products are cycled, recycled, and discarded.

The automatic calculation of exergy within the Aspen HYSYS^®^ environment has been accomplished. However, as [41] argues, the recalculation and extension of [52]’s RE is inefficient, as the properties and elements are already well-defined. As such, the RE is used as is. In order to calculate the exergy of the streams in the Aspen HYSYS^®^ GTL model, *user variables* were developed and employed. The following subsections explain how this was achieved.

#### 2.3.1. Physical Exergy

The physical exergy is the work available by taking a substance from its present state of *T* and *P* to the RE state of $T_{0}$ and $P_{0}$. The formula for calculating this for 1 mole of constituent is

(10) $$b_{ph}=\left(h-h_{0}\right)-T_{0}\left(s-s_{0}\right).$$

Implementing this calculation in Aspen HYSYS^®^ is a trivial matter, as shown in Algorithm 1.

The algorithm (implemented as an *user variable*) starts by obtaining the stream’s current enthalpy (*h*) and entropy (*s*). Next, the stream’s temperature and pressure are set to that of the RE (25 °C and 101.325 kPa). It is then forced to recalculate the stream’s enthalpy ($h_{0}$) and entropy ($s_{0}$). The physical exergy per mole is then computed by implementing (10) as is. In order to obtain the stream’s total physical exergy ($B_{ph}$), the per mole exergy is multiplied by the stream’s molar flow. To verify that the physical exergy was calculated correctly, it was compared to hand calculations as well as the integrated physical exergy in Aspen HYSYS^®^. It should be noted that the physical exergy is not dependent on the composition of the stream’s phases. As such the total stream’s physical exergy can be calculated.

**Algorithm 1** Calculation of physical exergy ($B_{ph}$)**Require:** Reference environment temperature $T_{ref}$ and pressure $P_{ref}$ in the simulation
  1:$Stream\leftarrow Stream_{Simulated}$  2:**if**Stream.VapourFractions.IsKnown**and**Stream.MolarFractions.IsKnown**and**Stream.MolarFlow.IsKnown**then**  3:    H←Stream.Enthalpy  4:    S←Stream.Entropy  5:    $Stream.Temperature\leftarrow T_{ref}$  6:    $Stream.Pressure\leftarrow P_{ref}$  7:    Stream.TPFlash()  8:    $H_{0}\leftarrow Stream.Enthalpy$  9:    $S_{0}\leftarrow Stream.Entropy$10:    $B_{ph}\leftarrow \left(H-H_{0}\right)-\left(T_{ref}+273.15\right)\left(S-S_{0}\right)$11:    F←Stream.MolarFlow.GetValue(“kgmole/h”)12:    $B_{ph}\leftarrow B_{ph}F$13:**end if**

#### 2.3.2. Chemical Exergy

Chemical exergy is the energy available to do work when a substance is brought from the RE state ($T_{0}$ and $P_{0}$) to a state of total thermodynamic equilibrium (dead state). There is more than one method that can be used to calculate the chemical exergy of a stream, as demonstrated by [41,53]. The approach followed, first proposed by [54] and implemented by [41], seems the most elegant and is accomplished by using

(11) $$b_{ch}=\sum x_{\left(i\right)}b_{ch(i)}^{0},$$

where $x_{\left(i\right)}$ is the mole fraction and $b_{ch(i)}^{0}$ is the standard molar chemical exergy of substance *i*. The $b_{ch(i)}^{0}$ values are defined by from the RE in [52]. In order to utilize (11), the standard molar chemical exergy of all the relevant substances is firstly made available to the *simulation basis* by creating a *user property* that tabulates the corresponding values. Not all substances’ standard chemical exergies are readily available though. Fortunately, any unknown constituents’ $b_{ch}^{0}$ can be calculated by making use of an appropriate reaction equation. The chosen reaction equation should contain only one of the unknown substances and all other known substances. For this study, some of the hydrocarbons’ standard chemical exergies were not available. Therefore, these had to be calculated by making use of the combustion equation:

(12) $$C_{\alpha }H_{\beta }+\left(\alpha +\frac{\beta }{4}\right)O_{2}\;\to \;\alpha CO_{2}+\frac{\beta }{2}H_{2}O\left(\ell \right),$$

with α and β coefficients corresponding to the considered hydrocarbon numbers. By modifying (12) considerably, as detailed in [45], the standard chemical exergy can be calculated by using

(13) $$\begin{array}{cc} b_{ch(i)}^{0}= & \left[{\bar{g}}_{\left(i\right)}^{0}+\left(\alpha +\frac{\beta }{4}\right){\bar{g}}_{\left(O_{2}\right)}^{0}-\alpha {\bar{g}}_{\left(CO_{2}\right)}^{0}-\frac{\beta }{2}{\bar{g}}_{\left(H_{2}O\left(\ell \right)\right)}^{0}\right]\\ & +\alpha b_{ch(CO_{2})}^{0}+\frac{\beta }{2}b_{ch(H_{2}O\left(\ell \right))}^{0}-\left(\alpha +\frac{\beta }{4}\right)b_{ch(O_{2})}^{0}. \end{array}$$

Equation (13) makes use of Gibbs’ function of formation and known standard chemical exergy values. The values used are summarized in Table 4. Finding tabulated Gibbs’ function of formation values for the hydrocarbons (${\bar{g}}_{\left(i\right)}^{0}$) proved difficult. By converting the values published in [55], the standard chemical exergy was calculated. Some of the calculated standard chemical exergy values were compared to known hydrocarbon values in order to verify whether they could be considered adequate. Table 5 shows the comparison of the values of CH_4_-C_5_H_12_. Based on the marginal differences seen, the calculated hydrocarbon exergies were deemed acceptable.

In order to calculate an Aspen HYSYS^®^ stream’s total chemical exergy, a new *user variable* was developed. Within Aspen HYSYS^®^, a *user variable* is a section of program codes developed by the user. Typically, a *user variable* can be connected to the entire simulation model, a specific component of, in this case, a specific material flow stream. For a GTL process, there will understandably be some multi-phase streams. Some substances, such as H_2_O, have different standard chemical exergy values when in different phases. To take this into account, the total chemical exergy was taken as the sum of the vapor, the liquid, and the aqueous phase exergy. Mathematically this is conveyed as

(14) $$\begin{array}{cc} b_{ch}= & \sum x_{(i)v}b_{ch(i)v}^{0}+\sum x_{(i)\ell }b_{ch(i)\ell }^{0}\\ & +\sum x_{(i)a}b_{ch(i)a}^{0}. \end{array}$$

The phase exergy was assumed to be zero whenever the phase was not present in the stream. Algorithmically, the process for calculating chemical exergy is outlined in Algorithm 2.

To verify whether the chemical exergy was calculated correctly, hand calculations were again used as a comparison. By implementing the *user variable* for all streams within the Aspen HYSYS^®^ model, the normal operating condition of the process was expressed in terms of physical and chemical exergies.

#### 2.3.3. Fault Conditions

To evaluate whether the exergy descriptions of the process could be used to detect faults, eleven fault conditions were identified. These faults were chosen in such a way as to appear in all of the critical process units and paths and also to excite expected loop phenomena. Faults were selected based on process knowledge and the sensitivities thereof.

When specifying the faults within the ATR section ($F_{1q}$), the following effects were of particular interest:

- The same type of fault of the same magnitude and location but in opposite directions. These faults are notated as $F_{11}$ and $F_{12}$ respectively and are representative of deviations in the feed molar flow rate.
- Two different types of faults of the same magnitude and location. This refers to $F_{12}$ and $F_{13}$. Here, fault $F_{13}$ is the result of the feed stream’s pressure being too low.
- The same type of fault of the same magnitude but slightly different locations, $F_{13}$ and $F_{14}$. Fault $F_{14}$ would represent fouling of the reactor bed.

**Algorithm 2** Calculation of chemical exergy ($B_{ch}$)**Require:** Components’ chemical exergies (per phase) defined in the simulation
  1:$Stream\leftarrow Stream_{Simulated}$  2:**if**(Stream.Pressure.IsKnownandStream.MolarFlow.IsKnownandStream.MolarFractions.IsKnownandStream.VapourFractions.IsKnownthen  3:    Components←Stream.Components  4:    $B_{ch}=0$  5:    **for** each Component **do**  6:        **if** Component’s molar flow > 0 **then**  7:           $m_{v}\leftarrow {\mathrm{Component}}^{\prime }\mathrm{svapormolarflow}$  8:           $m_{l}\leftarrow {\mathrm{Component}}^{\prime }\mathrm{slightliquidmolarflow}$  9:           $m_{a}\leftarrow {\mathrm{Component}}^{\prime }\mathrm{sheavyliquidmolarflow}$10:           $m_{T}\leftarrow {\mathrm{Component}}^{\prime }\mathrm{stotalmolarflow}$11:           $ratio_{v}=m_{v}/m_{T}$12:           $ratio_{l}=m_{l}/m_{T}$13:           $ratio_{a}=m_{a}/m_{T}$14:           $mF_{T}\leftarrow {\mathrm{Component}}^{\prime }\mathrm{stotalmolefraction}$15:           $mF_{v}=ratio_{v}*mF_{T}$16:           $mF_{v}=ratio_{l}*mF_{T}$17:           $mF_{v}=ratio_{a}*mF_{T}$18:           $B_{ch_{v}}=mF_{v}*B_{ch_{v}}*m_{v}$19:           $B_{ch_{l}}=mF_{l}*B_{ch_{l}}*m_{l}$20:           $B_{ch_{a}}=mF_{a}*B_{ch_{a}}*m_{a}$21:           $B_{ch_{component}}=B_{ch_{v}}+B_{ch_{l}}+B_{ch_{a}}$22:        **end if**23:        $B_{ch}=B_{ch}+B_{ch_{component}}$24:    **end for**25:**end if**


Similarly for the FTR section ($F_{2q}$), the subsequent faults were evaluated:

- As stated by [40], the Fischer–Tropsch process is sensitive to deviations in temperature. Fault $F_{21}$ is the result of insufficient heating of Heater 1, delivering reactor feed at a lower temperature. $F_{24}$ is representative of a problem regarding the water cooling of the reactor, causing the reaction temperature to increase.
- $F_{22}$ and $F_{23}$ are based on the notion that there could be damaged pipes, resulting in leakages and pressure drops.

Finally, the recycle section ($F_{3q}$) was subjected to the following faults:

- The recycle compressor’s efficiency could degrade over time, resulting in lower compression $F_{31}$ being achieved
- A blockage in the gas splitter $F_{32}$ results in less gas being recycled and a subsequent increase in purge gas volume.
- $F_{33}$ simulates the effect of a pipe leak in the recycle stream itself, the cause of which is largely of secondary concern.

**Figure 4:**
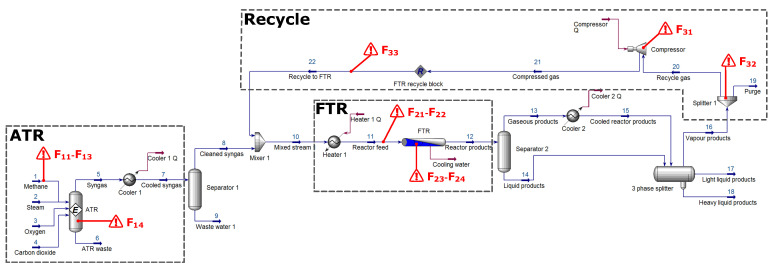
Aspen HYSYS^®^ GTL process flow diagram indicating the locations of simulated faults.

The main purpose of investigating faults within the recycle stream ($F_{3q}$) is to evaluate whether the fault location can be pinpointed or whether it will inevitably propagate throughout the entire process. The particular location of a fault is indicated with a red triangle on the HYSYS^®^ model in Figure 4.

In Table 6, the fault conditions and their specifics are summarized. The normal operating point and each of the fault conditions were simulated individually. This is in line with typical FDI literature that does not directly address the simultaneous occurrence of faults (so-called multiple faults).

Insofar as the effects of the faults are concerned, the following is of importance. Once a fault has been introduced, the simulated plant is allowed to reach steady -state before any analysis is conducted. Naturally, this would affect the produced products, or at the very least, the distribution of output products. Since the upgrading section was not considered in this study, the effects of the fault(s) on the output products were not specifically considered.

In a physical plant stochastic process, variations would naturally occur, in addition to any possible fault conditions. The methodology followed here assumes that the normal operating condition can be described by a constant steady state. Conceptually, then, this allows the FDI-scheme to be evaluated in terms of the absolute best-case scenario from an operational perspective.

### 2.4. Fault Detection and Isolation Methodology

**Figure 5:**
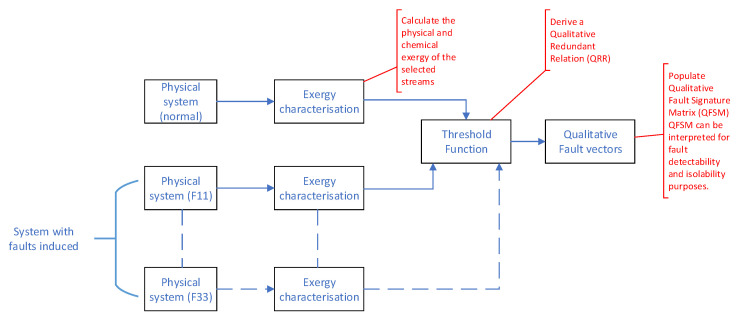
Graphical representation of the exergy-based FDI scheme.

The FDI methodology essentially compares the exergy relationships within the process plant between normal operating conditions (NOC), faulty operating conditions, and the current operating conditions in a qualitative fashion. In order to facilitate a detailed discussion of the methodology, refer to Figure 5.

**Figure 6:**
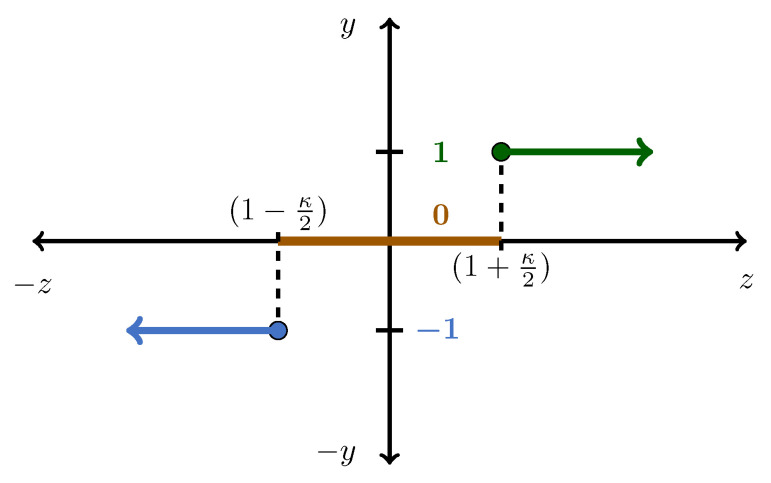
Graphical representation of a threshold function.

Initially the process was operated at NOC and the exergy characterization was performed (as detailed in Section 2.3). The data obtained from the exergy characterization phase were processed by means of a threshold function to derive a Qualitative Redundant Relation (QRR). A QRR is a vector that indicates the qualitative variation (positive, negative, or zero) of the parameters under consideration. In this case, the QRR would indicate variation in the exergy (both physical and chemical) between the current operating point and the NOC. The threshold function used to calculate the QRR had the form shown in Figure 6 and can be described by

(15) $$y=\left\{\begin{array}{cc} -1 & \mathrm{if}\;z<\left(1-\frac{\kappa }{2}\right)\\ 1 & \mathrm{if}\;z>\left(1+\frac{\kappa }{2}\right)\\ 0 & \mathrm{otherwise}, \end{array}\right.$$

where *z* represents the normalized exergy value under consideration and *y* is the magnitude of the resultant fault element. By applying the threshold function to the normalized data, a qualitative matrix was obtained with the form

(16) $$F_{pq}=\left[\begin{array}{cc} y(B_{ph(stream1)}) & y(B_{ch(stream1)})\\ \vdots & \vdots \\ y(B_{ph(stream22)}) & y(B_{ch(stream22)}) \end{array}\right]$$

To assign an appropriate value for $\frac{\kappa }{2}$, the variations within the HYSYS^®^ simulation executions were examined. Each time the model was simulated under identical conditions, small solver variations were seen. To ensure that simulation variations were not confused with faults, the simulation variances were quantified. The exergy values of the streams were recorded for four separate simulation runs and normalized accordingly. By employing the error analysis, as summarized in Table 7, a value of $\frac{\kappa }{2}=0.00635$ was selected. Essentially, the κ value determines a band in which any variation in the normalized exergy values are ignored. The latter effectively mitigates false positives in the FDI scheme that could be attributed to simulation variances.

For each of the identified fault conditions, $F_{11}$ through $F_{33}$, QRRs were developed according the the process followed for the NOC QRR. If the QRRs of the various faults were unknown, deviation from the current QRR to the NOC QRR would be indicative of a fault being present. However, should QRRs be available for each of the identified faults ($QRR_{11}$ through $QRR_{33}$), fault isolation could be accomplished by matching the current QRR with one of the identified faults.

Applying the threshold function with $\frac{\kappa }{s}=0.00635$ to the normalized simulation data resulted in tables of qualitative fault vectors.

## 3. Results and Discussion

In order to generate the set of results, the process plant was operated at a constant operating point (excluding the introduction of faults) with the various stream values depicted in Table 3. The simulated process environment was maintained at 25 °C and 1 atm.

The complete set of qualitative fault vectors obtained from the exergy-based FDI scheme is presented in Table 8. A fault was deemed *detectable* if the resultant qualitative fault vector was non-zero. By inspection, all of the identified faults were detectable. Thus, the exergy-based FDI scheme provides 100% detectability for PC processes of representative complexity.

In order for the faults to be *isolable*, no two faults should have identical fault vectors. By comparing the vectors of each fault to all others, the isolability performance of the fault detection scheme can be evaluated. By subtracting two fault vectors from each other, the distance between the vectors can be determined. For the 20×2 fault vectors in this case study, the distance between fault vectors *x* and *y* was defined as follows:

(17) $$d_{xy}=\sum_{j=1}^{2}\sum_{k=1}^{20}\left|F_{x}\left(k,j\right)-F_{y}\left(k,j\right)\right|.$$

$F_{x}\left(k,j\right)$ and $F_{y}\left(k,j\right)$ represent the entries in the respective fault vectors. By determining the distance between each fault vector and all the other vectors, an indication of the degree of isolability was obtained. Table 9 gives the isolability performance in the form of distances between the different fault vectors.

Zeros on the main diagonal only reveal 100% isolability performance. A zero implies zero distance and thus, a direct match between the respective fault vectors. In this case, with no zeros in the off-diagonal positions, no matches were identified between any of the fault vectors, implying 100% isolability.

When inspecting each column of the isolability matrix in Table 9, it is clear that the minimum distance of zero corresponds with the actual fault, as desired. Apart from the desired single zero per column, of significance is the next lowest distance, as reflected by the shortest distance to another fault. Upon inspecting each column, the shortest distance observed is 1 and that is between fault vectors $F_{13}$ and $F_{14}$. This reveals the weakest point in the isolability performance of the FDI scheme. This is conceptually supported, since any pressure drop in an input stream to the ATR will be reflected in the output pressure of the ATR. Similarly, if the pressure drop occurs inside the ATR (due to bed fouling for instance), the effect post-ATR will be very similar. If the exergy values of the constituent input streams were known, improved discrimination would be possible, for instance, allowing determination of which input stream’s pressure is too low.

The next smallest distance per column is 4 between fault vectors $F_{32}$ and $F_{33}$ and then 6 for the distance between fault vectors $F_{33}$ and $F_{22}$. Considering $F_{32}$ and $F_{33}$, the effect of the faults is very similar. For $F_{33}$, the amount of gas being recycled was reduced by 10% by means of a reduced split ratio, while for $F_{32}$, a leak of 10% was induced by means of an additional splitter. The two faults are not identical due to the location of the “leak”. Essentially the induced leak has a higher exergy destruction value than that caused by a splitter malfunction. Similar arguments hold for $F_{33}$ and $F_{22}$. The macro effect is the same, but the recycle stream leak is less costly from an exergy destruction perspective.

The largest distance between two fault vectors is 64 and that is between $F_{11}$ and $F_{12}$. Since the amount of methane present in the system directly affects the ratio of the syngas produced, and thus by extension, the quantity and quality of the product, good discrimination is to be expected. Excess methane is (to a degree) more acceptable than a shortage due to the effect of the recycle stream. However, any deviation would be detrimental to the overall efficiency of the process, and the proposed technique correctly identifies this.

Environmental effects (such as a change in temperature) were not considered explicitly in these results. However, since all exergy values are calculated based on a selected reference environment (SRE), environmental changes will affect all exergy values equally. Thus, even though changing environmental conditions will affect the numeric values obtained, the results will not be affected negatively.

Based on the results presented, the exergy-based FDI scheme has been shown to provide both a 100% detection rate and complete isolability performance for the identified faults. The GTL process considered in this work is considerably more complex than the ATR used by [19] and could be considered representative of a typical industrial process.

The performance of the proposed FDI scheme is surprising, given that it is fundamentally based on the threshold values of energy signals. However, it should be kept in mind that the sensitivity of the technique has not been verified against small incipient faults. The effects of sensor noise on the performance of the technique were also excluded in order to maintain the focus on the applicability of the technique for specific process configurations. However, the current set of faults can, to a large degree, be considered significant process faults without leading to catastrophic process failure.

## 4. Conclusions

The results suggest that the exergy-based FDI scheme performs well when applied to PC processes of representative complexity. The implementation presented here is an agnostic process which would allow rapid application to other processes modeled in Aspen HYSYS^®^. Further work should comprise a detailed analysis of the exergy-based technique’s performance in noisy environments, a sensitivity analysis with regard to the magnitude of detectable faults, and the assessment of the performance of the technique in a dynamic simulation environment. Given the sensitivity of the technique to the environmental conditions (assumed to be static in this work), the effect of a dynamically changing environment needs to be investigated. Additionally, a comparison between the exergy-based scheme and other common FDI techniques should also be considered.

## Figures and Tables

**Table 1:** Summary of the auto thermal reformer (ATR) feed stream ratios and molar flow rates.

Ratio			Molar Flow Rates [kgmole/h]		
H_2_O/C	=	0.6625	H_2_O	=	5429.2
O2/C	=	0.5412	O2	=	4435.5
CO_2_/C	=	0.1171	CO_2_	=	959.8

**Table 2:** The kinetic values used for the reactions in HYSYS.

Parameter	Arrhenius Expression		Unit
	A	E	
$k_{1}$	$8.8\times 10^{-6}$	37326	$\frac{{\mathrm{kgmole}}_{{\mathrm{CH}}_{4}}}{{\mathrm{Pa}}^{1.05}m^{3}\cdot s}$
$K_{1}$	$1.1\times 10^{-12}$	−68401.5	${\mathrm{Pa}}^{-1}$
$k_{2}$	$1.6\times 10^{-5}$	37326	$\frac{{\mathrm{kgmole}}_{\mathrm{CO}}}{{\mathrm{Pa}}^{1.25}m^{3}\cdot s}$

**Table 3:** Stream information of the GTL process.

Section	Stream No.	Description	Temperature (°C)	Molar Flow (kgmole/h)	Pressure (kPa)
ATR	1	Methane	675	8195.0	3000
	2	Steam	675	5429.2	3000
	3	Oxygen	200	4435.5	3000
	4	Carbon dioxide	675	959.8	3000
	5	Syngas	1029	30,262.6	3000
FTR	7	Cooled syngas	38	30,262.6	3000
	8	Cleaned syngas	38	24,452.7	3000
	9	Waste water 1	38	5810.0	3000
	10	Mixed stream 1	54	34,310.3	3000
	11	Reactor feed	210	34,310.3	2000
	12	Reactor products	213	22,290.4	1940
	13	Gaseous products	213	22,238.3	1940
	14	Liquid products	213	52.1	1940
	15	Cooled reactor products	38	22238.3	1940
	16	Vapour products	44	16,063.3	1940
	17	Light liquid products	44	189.8	1940
	18	Heavy liquid products	44	6037.3	1940
Recycle	19	Purge	44	3212.7	1940
	20	Recycle gas	44	12,850.6	1940
	21	Compressed gas	88	12,850.6	3000
	22	Recycle to FTR	88	9857.7	3000

**Table 4:** Values used to calculate the standard chemical exergies of the unknown hydrocarbons.

${\bar{g}}_{\left(O_{2}\right)}^{0}$	${\bar{g}}_{\left({\mathrm{CO}}_{2}\right)}^{0}$	${\bar{g}}_{\left(H_{2}O\left(\ell \right)\right)}^{0}$	$b_{\mathrm{ch}({\mathrm{CO}}_{2})}^{0}$	$b_{\mathrm{ch}(H_{2}O\left(\ell \right))}^{0}$	$b_{\mathrm{ch}(O_{2})}^{0}$
0	−394,360	−237,180	19,480	900	3970

**Table 5:** Comparing tabulated and calculated standard chemical exergies of known hydrocarbons.

Substance	Tabulated	Calculated	Difference [%]
CH_4_	831,200	831,275	0.009
C_2_H_6_	1,495,000	1,495,144	0.010
C_3_H_8_	2,152,800	2,150,505	0.107
C_4_H_10_	2,804,200	2,804,251	0.002
C_5_H_12_	3,461,300	3,457,721	0.103

**Table 6:** The location, description, and details of the simulated faults.

Fault ID	Location	Description	Details
$F_{1q}$	**ATR section**		
$F_{11}$	Methane stream	Molar flow +10%	+819.5 kgmole/h
$F_{12}$	Methane stream	Molar flow −10%	−819.5 kgmole/h
$F_{13}$	Methane stream	Pressure −10%	−300 kPa
$F_{14}$	ATR	Pressure −10%	−300 kPa
$F_{2q}$	**FTR section**		
$F_{21}$	Reactor feed stream	Temperature −10%	−21 °C
$F_{22}$	Reactor feed stream	Leakage −10%	Splitter 0.9:0.1
$F_{23}$	FTR	Pressure −10%	−200 kPa
$F_{24}$	FTR	Temperature −10%	−1.13E+08 kJ/h
$F_{3q}$	**Recycle section**		
$F_{31}$	Compressor	Pressure −10%	−300 kPa
$F_{32}$	Splitter 1	Lower split ratio	0.700:0.300
$F_{33}$	Recycle to FTR	Leakage −10%	Splitter 0.9:0.1

$F_{pq}$—where *p* represents the section and *q* the fault number.

**Table 7:** Calculating the threshold value $\frac{\kappa }{2}$.

Parameters		$B_{\mathrm{ph}}+B_{\mathrm{ch}}$
Confidence level	CL	95
Number of samples	*m*	10
Degrees of freedom	m−1	9
Average	${\bar{B}}_{\_h}$	1.0010
Standard deviation	*s*	0.0021
t-value	$t_{m-1}$	3.69
Error	$t_{m-1}\frac{s}{\sqrt{m}}$	0.0024

**Table 8:** Qualitative fault vectors for each of the identified faults

Stream no	F1q								F2q								F3q					
	F11		F12		F13		F14		F21		F22		F23		F24		F31		F32		F33	
	Bph	Bch	Bph	Bch	Bph	Bch	Bph	Bch	Bph	Bch	Bph	Bch	Bph	Bch	Bph	Bch	Bph	Bch	Bph	Bch	Bph	Bch
1	1	1	−1	−1	−1	0	0	0	0	0	0	0	0	0	0	0	0	0	0	0	0	0
2	0	0	0	0	0	0	0	0	0	0	0	0	0	0	0	0	0	0	0	0	0	0
3	0	0	0	0	0	0	0	0	0	0	0	0	0	0	0	0	0	0	0	0	0	0
4	0	0	0	0	0	0	0	0	0	0	0	0	0	0	0	0	0	0	0	0	0	0
5	−1	1	1	−1	−1	0	−1	0	0	0	0	0	0	0	0	0	0	0	0	0	0	0
7	1	1	−1	−1	0	0	0	0	0	0	0	0	0	0	0	0	0	0	0	0	0	0
8	1	1	−1	−1	0	0	0	0	0	0	0	0	0	0	0	0	0	0	0	0	0	0
10	1	1	−1	−1	0	0	0	0	0	0	−1	−1	0	0	0	0	−1	0	−1	−1	−1	−1
11	1	1	−1	−1	0	0	0	0	−1	0	−1	−1	0	0	0	0	0	0	−1	-1	−1	−1
12	1	1	−1	−1	1	0	1	0	−1	0	−1	−1	−1	0	1	0	0	0	−1	−1	−1	−1
13	1	1	−1	−1	1	0	1	0	−1	−1	−1	−1	−1	0	1	1	0	0	−1	−1	−1	−1
14	1	−1	1	1	1	−1	1	−1	−1	1	1	1	−1	−1	−1	−1	0	0	−1	−1	−1	−1
15	1	1	−1	-1	0	0	0	0	0	−1	−1	−1	−1	0	0	1	0	0	−1	−1	−1	−1
16	1	1	−1	−1	0	0	0	0	0	0	−1	−1	−1	0	0	0	0	0	−1	−1	−1	−1
17	−1	−1	1	−1	1	0	1	0	−1	−1	1	−1	−1	0	−1	1	0	0	−1	−1	−1	−1
18	−1	1	1	−1	1	0	1	0	−1	0	1	−1	−1	0	−1	0	0	0	−1	−1	−1	−1
19	1	1	−1	−1	0	0	0	0	0	0	−1	−1	−1	0	0	0	0	0	1	1	−1	−1
20	1	1	−1	−1	0	0	0	0	0	0	−1	−1	−1	0	0	0	0	0	−1	−1	−1	−1
21	1	1	−1	−1	0	0	0	0	0	0	−1	−1	1	0	−1	0	−1	0	−1	−1	−1	−1
22	1	1	−1	−1	0	0	0	0	0	0	−1	−1	1	0	−1	0	−1	0	−1	−1	−1	−1

**Table 9:** Isolability performance based on the separation distance metric.

	$F_{11}$	$F_{12}$	$F_{13}$	$F_{14}$	$F_{21}$	$F_{22}$	$F_{23}$	$F_{24}$	$F_{31}$	$F_{32}$	$F_{33}$
$F_{11}$	0	64	32	31	38	54	36	31	37	48	52
$F_{12}$	64	0	34	35	30	10	34	41	31	20	16
$F_{13}$	32	34	0	1	18	30	18	13	11	32	32
$F_{14}$	31	35	1	0	17	29	17	12	10	31	31
$F_{21}$	38	30	18	17	0	20	12	15	13	18	18
$F_{22}$	54	10	30	29	20	0	24	31	23	10	6
$F_{23}$	36	34	18	17	12	24	0	15	15	20	18
$F_{24}$	31	41	13	12	15	31	15	0	10	25	25
$F_{31}$	37	31	11	10	13	23	15	10	0	23	23
$F_{32}$	48	20	32	31	18	10	20	25	23	0	4
$F_{33}$	52	16	32	31	18	6	18	25	23	4	0
